# Spatio-Temporal Distribution Patterns in Environmental Factors, Chlorophyll-a and Microcystins in a Large Shallow Lake, Lake Taihu, China

**DOI:** 10.3390/ijerph110505155

**Published:** 2014-05-14

**Authors:** Rui Ye, Kun Shan, Hailong Gao, Ruibin Zhang, Wen Xiong, Yulei Wang, Xin Qian

**Affiliations:** 1 State Key Laboratory of Pollution Control and Resource Reuse, School of the Environment, Nanjing University, Nanjing 210023, China; E-Mails: rui.ye0220@163.com (R.Y.); NJUGHL@gmail.com (H.G.); zhangrb88@126.com (R.Z.); njuxiongwen@163.com (W.X.); gywyl127@163.com (Y.W.); 2 State Key Laboratory of Freshwater Ecology and Biotechnology, Institute of Hydrobiology, Chinese Academy of Sciences, Wuhan 430072, China; E-Mail: shankun@sina.com; 3 University of Chinese Academy of Sciences, Beijing 100049, China

**Keywords:** eutrophication, Lake Taihu, stratification, spatial-temporal distribution, microcystin-LR, microcystin-RR

## Abstract

The spatio-temporal distribution of environmental factors, chlorophyll-a (Chl-a), and microcystins (MCs) in a shallow lake, Lake Taihu (China), were investigated from 2009 to 2011 on a monthly basis at nine sampling stations. The annual mean concentration ranges of total nitrogen (TN), total phosphorus (TP), Chl-a, MC-LR and MC-RR were 0.17–10.53 mg/L, 0.027–0.581 mg/L, 0.10–129.75 µg/L, 0.013–2.019 µg/L and 0.002–0.794 µg/L, respectively. The average TN, ammonium (NH_4_^+^) and TP concentrations in Meiliang Bay decreased from 3.54 to 2.26 mg/L, 0.63 to 0.31 mg/L and 0.150 to 0.124 mg/L, respectively, when compared with values from 2006–2008, indicating that water quality has improved in severe cyanobacterial bloom areas in recent years. Additionally, the distribution of MCs was northern lake areas > western lake areas > central lake areas > macrophyte-dominated areas. Correlation analysis revealed that nutrients were the most important variable accounting for the variation of extracellular MC-LR concentration in heavy cyanobacterial bloom areas of Lake Taihu. During the study period, the maximum MCs concentration reached 2.75 ± 0.27 μg/L in the bloom period in the northern lake areas, which is more than two times the safety limit of 1 μg/L MCs required for drinking water. However, microcystins decreased gradually as the water quality improved from 2009 to 2011, indicating that the risk of MCs exposure was slightly decreased in Lake Taihu.

## 1. Introduction

Eutrophication in inland freshwater lakes is a worldwide environmental problem [1], and the mechanisms involved in the formation of eutrophication are of great concern. Nutrient overenrichment of water from agriculture, industrial waste and sewage has advanced the growth of cyanobacteria as harmful algal blooms [2,3,4].

Harmful cyanobacterial blooms cause deterioration of water quality and endanger the safety of aquatic ecosystems by releasing microcystins (MCs) from *Microcystis*, *Anabaena* and *Oscillatoria* [5]. MCs are monocyclic heptapeptide compounds with many different isomers [6,7], among which MC-LR, MC-RR and MC-YR are the dominant types. MC-LR and MC-RR have been selected as the main research objects because they account for a high proportion of MCs found in aquatic systems [8]. Microcystin toxicities resulting in human illnesses, as well as wildlife and fish kills, have been reported [7,9,10,11,12,13].

Lake Taihu is the third largest freshwater lake in China and one of the primary sources of drinking water in the region. However, Lake Taihu is also one of the most severely polluted freshwater lakes in China. Accordingly, cyanobacterial blooms in the lake have been investigated extensively [14,15,16,17,18,19,20]. A water supply crisis occurred in Wuxi in the summer of 2007 due to an algal bloom in Lake Taihu [21]. Since this event, many studies have been conducted to improve water quality and control eutrophication in Lake Taihu [22,23,24,25].

Lake Taihu is a large shallow lake with a unique shape and strong spatial heterogeneity of water quality and phytoplankton in different lake regions that each has different social and ecological functions. In recent years, most studies of Lake Taihu have focused on investigating nutrients and phytoplankton in individual bays or the center of the lake [26,27,28,29,30]. However, there is relatively little information available from interannual monitoring at the whole lake scale and simultaneously quantitative comparison with different sites based on the horizontal and vertical distribution of nutrients and phytoplankton.

In the present study, the monthly concentration of various nitrogen and phosphorus forms, Chl-a and microcystins in Lake Taihu was measured at nine sites for roughly three years from 2009 to 2011. The data were then analyzed for temporal and spatial (horizontal and vertical) patterns.

## 2. Materials and Methods

### 2.1. Study Area

**Figure 1:**
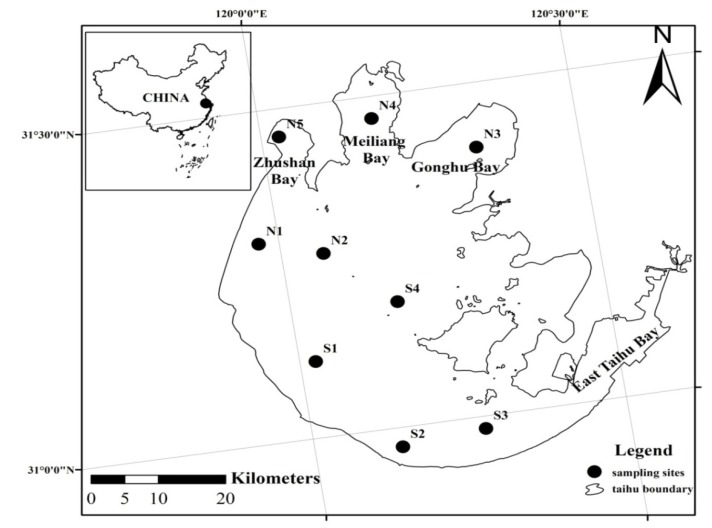
Sampling stations in Lake Taihu (N1–N5, S1–S4).

Lake Taihu (30°55'40''–31°32'58'' E, 119°52'32''–120°36'10'' N) is located in the lower Yangtze River Delta. The total surface area of the lake is 2,338 km^2^, with an average depth of only 1.9 m [31]. Lake Taihu is situated in a temperate and subtropical zone, with a humid and semi-humid monsoon climate. The annual average temperature in the region is 13–16 °C, with temperatures of 2–4 °C in January and 26–29 °C in July. The annual average precipitation is approximately 1,000–1,300 mm [32]. There is a clear distinction among the four seasons in Lake Taihu area. Spring, summer, autumn and winter in the lake area represent March–May, June–August, September–November, and December–February, respectively. In the present study, we selected nine sampling stations in the lake according to its limnological and geographical characteristics. These stations were located in the west lake zone (N1(31°18'5.80'' N, 119°58′3.40'' E), S1(31°6'55.49'' N, 120°1'30.34'' E)), central lake zone (N2(31°16'30.20'' N, 120°3'59.90'' E), S4(31°11'18'' N, 120°10'9'' E)), east lake zone (S3(30°58'53'' N, 120°16'21'' E)), south lake zone (S2(30°58'14.37'' N, 120°8'16.40'' E)), Gonghu Bay (N3(31°24'10.07'' N, 120°20'6.85'' E)), Meiliang Bay (N4(31°27'59.52'' N, 120°10'43.96'' E)) and Zhushan Bay (N5(31°27'26.87'' N, 120°1'42.29'' E)) (Figure 1).

### 2.2. Sampling and Data Measurements

Monthly surveys were conducted at each site from February 2009 to December 2011, except during June, October, and December of 2009 and February of 2010, near the end of each month. Water depth (WD), water temperature (WT), dissolved oxygen (DO), turbidity, and chlorophyll-a (Chl-a) profiles were measured throughout the water column at each site using a multi-parameter water quality sensor AAQ-1183 (Alec Electronics Co., Kobe, Japan). At each sampling site, temperature, DO, turbidity and Chl-a were monitored from the upper water column (0.2 m below water surface) to near-bottom waters (0.2 m above the bottom sediments) at a 0.5 m interval. Prior to deployment, the DO sensor was calibrated and its accuracy was confirmed to be within an average error of 1.5%.

The monthly physical, chemical, and biological data available for the nine sites during the monitoring period were also analyzed. Integrated water samples were collected using a 2 m long, 0.1 m wide handmade plastic tube with a one-way valve at the upper part of the tube. Sampling was conducted by collecting surface water from approximately 0.2 m below the water surface and bottom water from about 0.2 m above the bottom sediments with a columnar plexiglass water sampler at two sites (N1, N2) every month during the study period. Additionally, mixed samples of surface water and bottom water from the seven other sites were collected each month.

Total nitrogen (TN), total phosphorus (TP), nitrate (NO_3_^−^), ammonium (NH_4_^+^), nitrite (NO_2_^−^), total dissolved phosphorus (TDP), soluble reactive phosphorus (SRP), and suspended solids (SS) were analyzed based on standard methods [33]. TIN was defined as the sum of NO_3_^−^, NH_4_^+^, and NO_2_^−^. Chlorophyll a (Chl-a) concentrations were measured spectrophotometrically after extraction in 90% acetone [33].

For microcystins analysis, duplicate lake water samples were stored in 1,000 mL polyethylene bottles at 4 °C until transport to the laboratory for further analysis within 24 h. These samples were filtered through a glass microfiber filter (Whatman GF/C), after which the filtrate was cleaned using a Sep-Pak cartridge that was subsequently eluted with 10 mL pure water and 20% (*v/v*) aqueous methanol. The eluted solution was then evaporated to dryness by nitrogen and the residue was dissolved in 50% aqueous methanol for the subsequent HPLC assays [34]. For HPLC analysis, the column temperature was maintained at 40 °C and the injection volume was 20 μL. Samples were analyzed by applying 57% solution A (100% methanol) and 43% solution B (0.05 M KH_2_PO_4_, pH = 3) to the column over 20 min at a flow rate of 1 mL/min [35] and then comparing the results to those observed upon analysis of microcystins standards (MC-RR, YR, LR) obtained from Institute of Hydrobiology, Chinese Academy of Sciences (IHB-CAS). In the present study, MC-YR was contributed a very low proportion in comparison to the levels of MC-LR and MC-RR. Therefore, MC-LR and MC-RR have been selected as the main research objects.

### 2.3. Statistical Analysis

Two-tailed Pearson’s correlation analysis was used to determine the relationships between MCs and environmental factors [36]. The data distribution over time and relevant water quality parameters were presented using boxplots showing the median, 25th and 75th percentiles [37]. The bottom and top of the box are the first and third quartiles, and the band inside the box is the median. The “whisker” above and below the box represents the maximum and minimum of all of the data. The cross (“+”) represents outliers which are not included between the whiskers.

## 3. Results and Discussion

### 3.1. Temporal Changes in Nutrients and Chl-a in Lake Taihu

**Figure 2:**
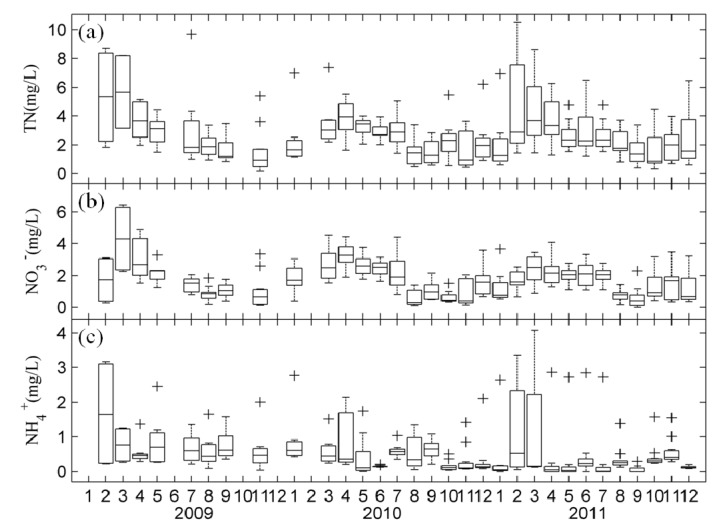
Monthly variability of TN, NO_3_^−^ and NH_4_^+^ in Lake Taihu: (**a**) TN; (**b**) NO_3_^−^; (**c**) NH_4_^+^ *boxplot* based on nine sites. The crosses “+” represent outliers.

Monitoring revealed strong seasonal variation in the major nutrient and Chl-a levels among study sites. Seasonality analysis of samples collected during 2009, 2010, and 2011 showed that TN was present at the highest levels in late spring and early summer (1.30–10.53 mg/L) and the lowest levels in autumn (0.34–5.47 mg/L) (Figure 2a). Similar to TN, NO_3_^−^ peaked in spring (0.27–6.44 mg/L) and then rapidly decreased in autumn (0.01–3.19 mg/L) (Figure 2b,c). However, there were no apparent temporal trends in NH_4_^+^. The fluctuation in NH_4_^+^ was not strong, and it remained at a relatively low level (0.52 ± 0.66 mg/L) throughout the observation period. The highest levels of Chl-a and SRP, which were directly available to algae, were observed in summer at ranges of 0.74–129.76 μg/L and 0.001–0.107 μg/L, respectively. In addition, the highest levels of TP were observed in summer at ranges of 0.027–0.581 mg/L, which may be caused by abundant phosphorus release from the sediment in summer. Meanwhile, there was a second-highest level (0.027–0.552 mg/L) appeared in winter and spring, which may be related to the non-point source phosphorus load in the flood season.

**Figure 3:**
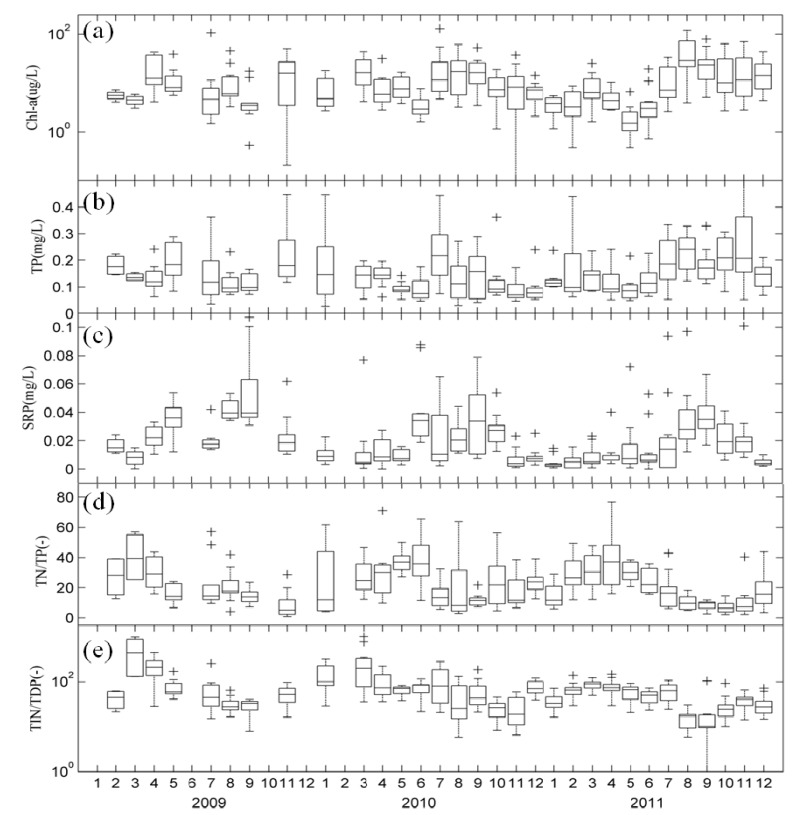
Monthly variability of Chl-a, TP, SRP, TN/TP and TIN/TDP in Lake Taihu: (**a**) Chl-a; (**b**) TP; (**c**) SRP; (**d**) TN/TP; and (**e**) TIN/TDP *boxplot* for nine sampling sites. The crosses “+” represent outliers.

Our monitoring data showed that the TN/TP ratios during winter and spring were higher than in summer (Figure 3d), which reflected a seasonal pattern driven by out-of-step dynamics of TN and TP in Lake Taihu. During the winter–spring period, inflowing rivers carried a larger amount of nitrogen into Lake Taihu, which led to a high TN/TP ratio, while in summer, low TN/TP ratios were caused by dominant cyanobacterial blooms [38]. During the blooms, the catabolism of bottom dead cyanobacteria and detritus organic materials consumed high levels of dissolved oxygen, facilitating the release of phosphorus from sediments and reducing the TN/TP ratio. In addition, TIN/TDP mass ratios revealed a seasonal dynamic analogous to TN/TP ratios (Figure 3e).

Evaluation of the interannual variation revealed that concentrations of TN, TP, and Chl-a in all water samples ranged from 0.17 to 10.53 mg/L, 0.027 to 0.581 mg/L, and 0.10 to 129.75 µg/L, respectively. Box plots indicated that various forms of nitrogen decreased annually, but there was no significant change in phosphorus or Chl-a (Figure 2 and Figure 3).

Since the drinking water crisis of 2007, the Chinese government has implemented various measures to improve the ecological environment of Lake Taihu. For instance, the pollution discharged into the lake from rivers in the basin has been reduced, and massive ecological dredging has been employed in Gonghu Bay, Meiliang Bay and Zhushan Bay. In addition, a water diversion project from Changjiang River to Lake Taihu was conducted to mitigate algal blooms in summer.

Xu *et al.* [23] reported the dynamic changes in monthly water quality in Meiliang Bay and the central portion of the lake from 2006 through 2008. In the present study, based on the approximate sampling locations, sampling date and analytical methods, we compared major nutrient indexes from 2009 to 2011 with those from three years before based on the mean value. The average TN concentration in Meiliang Bay decreased from 3.54 to 2.26 mg/L, while the average ammonium level fell from 0.63 to 0.31 mg/L at the two stations and the average TP concentration decreased from 0.150 to 0.124 mg/L in Meiliang Bay. Overall, these findings indicate that the main nutrients of the major lake areas have been declining in recent years. These results complement long-term studies of Lake Taihu, and provide a scientific foundation for further investigation of the lake.

### 3.2. Spatial Distribution of Major Environmental Factors and Chl-a in Lake Taihu

Lake Taihu is well mixed during most of the year because it is very shallow (the average depth is 1.9 m) and it is quite sensitive to wind disturbance. However, real-time monitoring conducted *in situ* found that some environmental factors appeared obvious vertical stratification under the meteorological conditions at some sampling sites (N1, N4 and N5).

**Figure 4:**
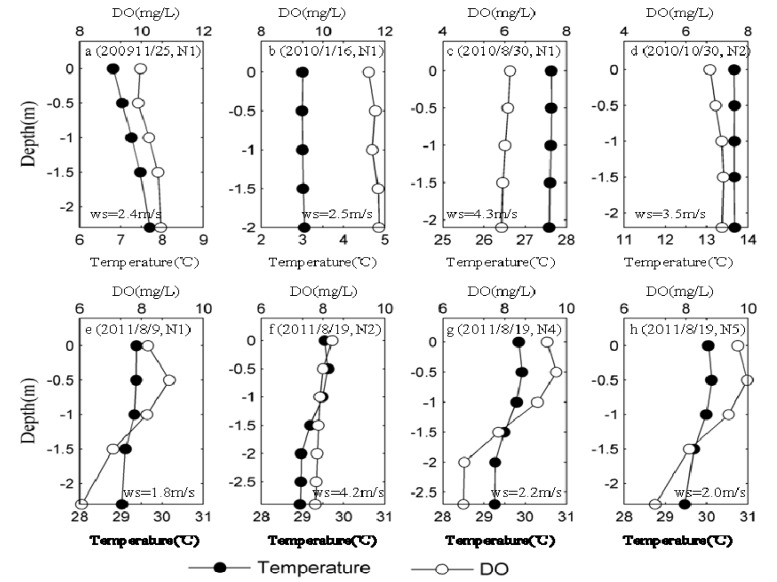
Profiles of temperature and DO in the lacustrine zone (N1-N5): (**a**) 2009/11/25 at N1; (**b**) 2010/1/16 at N1; (**c**) 2010/8/30 at N1; (**d**) 2010/10/30 at N2; (**e**) 2011/8/9 at N1; (**f**) 2011/8/19 at N2; (**g**) 2011/8/19 at N4; (**h**) 2011/8/19 at N5. “ws” represents the mean wind speed during the study period.

Temperature was found to be the main factor controlling lake stratification, which affects hydrodynamics via the formation of vertical density gradients [39]. Monitoring results revealed that the difference in water temperature was approximately within 1 °C in the vertical section, suggesting that Lake Taihu is not strongly thermally stratified. Real-time monitoring conducted in the field in August showed that the surface was only slightly warmer (maximum variance up to 0.6 °C) than the bottom at each site (Figure 4g) during summer. Surface water was about 0.9 °C cooler than bottom water at N1 station during fall through winter (Figure 4a), which was most likely due to rapid cooling associated with cold air. DO concentrations in the vertical section ranged from 5.9 to 13.7 mg O_2_/L in surface water and from 5.0 to 11.2 mg O_2_/L in bottom water, and hypoxic events were not observed during our monitoring period.

**Figure 5:**
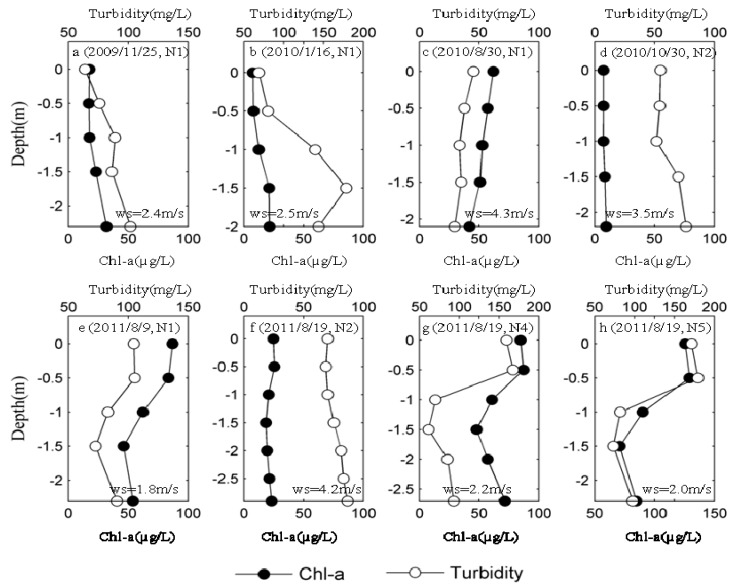
Profiles of turbidity and Chl-a in the lacustrine zone (N1-N5): (**a**) 2009/11/25 at N1; (**b**) 2010/1/16 at N1; (**c**) 2010/8/30 at N1; (**d**) 2010/10/30 at N2; (**e**) 2011/8/9 at N1 (**f**) 2011/8/19 at N2; (**g**) 2011/8/19 at N4; (**h**) 2011/8/19 at N5. “ws” represents the mean wind speed during the study period.

In most of the year, DO, Chl-a and turbidity did not differ significantly in the vertical section (Figure 5c,f), which may have been the result of high wind speed (Figure 4 and Figure 5). Lake Taihu generally presents a mixed state in the vertical section in cases in which wind speed is higher than the critical wind speed (about 3 m/s) for surface bloom formation [40].

Generally, the lowest DO concentrations are in accordance with periods of maximum water temperature in summer. However, in the period of the cyanobacterial bloom, relatively high DO concentrations were also observed in the surface water (approximately 0.5 m below water surface) of N1 (8.6 ± 0.3 mg O_2_/L, *n* = 5), N4 (9.5 ± 0.2 mg O_2_/L, *n* = 5) and N5 (9.8 ± 0.2 mg O_2_/L, *n* = 5), respectively (Figure 4e,g,h). This most likely occurred because a high abundance of *Microcystis* in the water via buoyancy regulation to optimize utilization of the nutrient and light resources, as indicated by the high Chl-a (above 80 µg/L) and turbidity (above 100 mg/L) in the surface water (Figure 5e,g,h). Therefore, *in situ* real-time investigation of the distribution of environmental factors in the vertical section of Lake Taihu can facilitate to describe the process by which surface cyanobacterial bloom form.

Additionally, real-time monitoring data also showed that Chl-a in bottom water is slightly higher than that in surface water from fall through winter, which serves as indirect evidence that sedimentation of Cyanobacteria during dormancy resulted in its accumulation into the sediment (Figure 5a,b).

**Figure 6:**
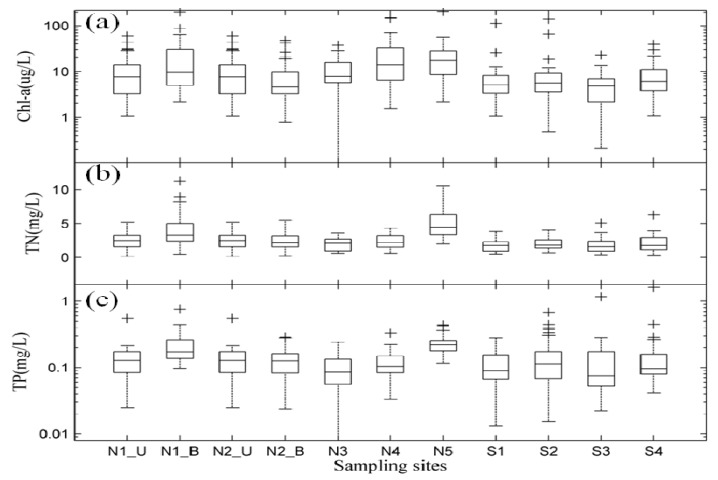
Spatial distribution of TN, TP and Chl-a in Lake Taihu based on monthly data. (**a**) Chl-a; (**b**) TN; and (**c**) TP. N1_U: surface of station N1; N1_B: bottom of station N1; N2_U: surface of station N2; N2_B: bottom of station N2. *boxplot* based on nine sampling sites. The cross “+” represents outliers.

Monitoring data from the nine sites showed that major nutrients and Chl-a had strong horizontal spatial variation in different lake zones. The largest concentrations of Chl-a, TN and TP located at Zhushan Bay were 25.20 ± 37.35 µg/L, 4.93 ± 2.22 mg/L and 0.232 ± 0.081 mg/L, respectively. Lower concentrations of Chl-a, TN and TP were located in Meiliang Bay (N4), the western lake zone (N1, S1), center lake zone (N2, S4), Gonghu Bay (N3) and southern lake zone (S2). Finally, the minimum concentrations of Chl-a, TN and TP located in the eastern lake zone were 5.40 ± 4.81 µg/L, 1.83 ± 1.09 mg/L, and 0.151 ± 0.213 mg/L, respectively (Figure 6).

In addition, Chl-a, TN and TP were measured at the surface and bottom of the western lake zone (N1) and at the center of the lake (N2). We found that the vertical distributions presented the opposite characteristics in N1 and N2. Specifically, the concentrations of Chl-a, TN and TP in surface water (N1_U) were lower than in bottom water (N1_B) at N1, while for N2 their concentrations were higher in surface water (N2_U) than in bottom water (N2_B) (Figure 6). N1 was located close to Dapu Harbor, which is considered the most polluted area in Lake Taihu and therefore has a large amount of nutrient-rich soft sludge in the bottom. N2 is located in the center of the lake, where little soft sludge was observed, which may be the main reason for the spatial differentiation between the two sampling sites. Overall, our measured data differed significantly in Lake Taihu, and it was found to have unique horizontal and vertical features.

### 3.3. Spatio-Temporal Distribution of MCs in Lake Taihu

**Figure 7:**
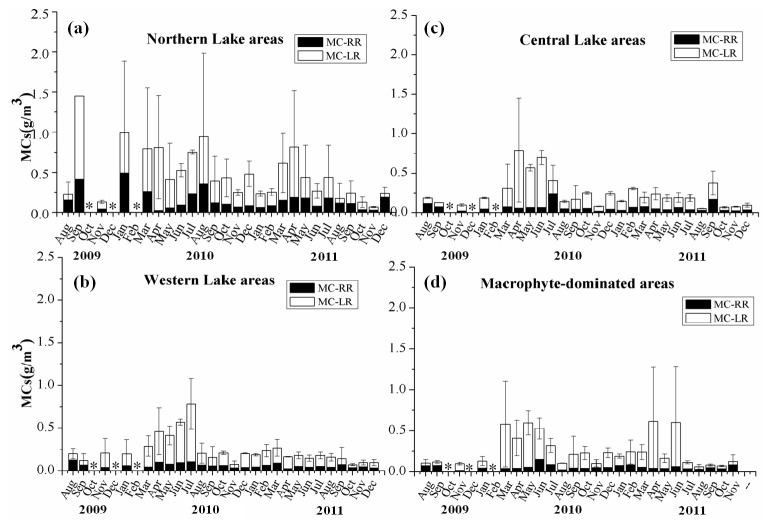
Monthly variability of MC-RR and MC-LR in different lake zones: (**a**) northern lake areas; (**b**) western lake areas; (**c**) central lake areas; (**d**) macrophyte -dominated areas. The asterisk“∗” represents missing sampling points that weren’t sampled in the date.

In this study we divided the lake into the following four zones to enable convenient quantitative comparison based on the dominant algae and macrophytes of the lake zone [41,42,43], geographical location of the sampling sites and nutrient and chlorophyll a levels at the sites: northern lake areas (N4, N5); western lake areas (N1, S1); macrophyte-dominated areas (S3, N3); central lake areas (N2, S4) (Figure 7). The concentration of MCs in different lake zones showed a clear spatial distribution pattern during the study period, occurring in the order northern lake areas (N4, N5) > western lake areas (N1, S1) > central lake areas (N2, S4) > macrophyte-dominated areas (S3, N3). Quantitative comparison among the four lake areas revealed that the maximum MCs concentration during the bloom period reached 2.75 ± 0.27 μg/L on Sep 25, 2009 in northern lake areas (Figure 7), which is more than two times the limit of 1 μg/L MCs required for drinking water [9].

We also detected temporal variations in the concentrations of MCs from 2009 to 2011. Interannual variation showed that the concentrations of MC-RR and MC-LR in all water samples ranged from 0.002 to 0.794 µg/L and 0.013 to 2.019 µg/L, respectively. The monitoring data indicated that MC-LR was higher than MC-RR during most of the monitoring period, and that the highest concentrations were observed in late spring and summer (Figure 7).

The microcystins decreased gradually as the water quality improved from 2009 to 2011 (Figure 7), which may be related to numerous restoration measures implemented in recent years. Based on the analysis of whole lake areas, relative high concentrations of MC-LR (0.270 ± 0.316 µg/L) and MC-RR (0.142 ± 0.176 µg/L) were observed in 2009–2010, respectively, while there is an obvious decline in 2011 (0.142 ± 0.176 µg/L, 0.066 ± 0.061 µg/L respectively) (Figure 7). The monitoring results also showed that the proportion of MC-RR increased clearly in MCs with the significant reduction of MC-LR concentration in study period (Figure 7). The toxicity of MC-LR is about five times greater than that of MC-RR [44]; therefore, these findings indicate that the risk associated with MCs exposure was slightly decreased in Lake Taihu.

### 3.4. Correlations between Extracellular MCs and Environmental Factors

Environmental factors associated with the concentration of extracellular MCs were investigated by correlation analysis (Table 1.) using the data monitored during the period of heavy cyanobacterial blooms (from June to October) in three years.

**Table 1:** Correlation coefficient between environmental variables in Lake Taihu (Pearson, 2-tailed, * for *p* < 0.05, significant; ** for *p* < 0.01, highly significant. LR/RR represents MC-LR/MC-RR.)

**Study Area**	**Northern Lake Area **(*n* = 27)			**Western Lake Area **(*n* = 39)		
**Variables**	**MC-LR**	**MC-RR**	**LR/RR**	**MC-LR**	**MC-RR**	**LR/RR**
TN	0.029	0.086	0.392 *	0.419 **	−0.021	0.447 **
NO_3_^−^	0.384	0.353	0.497 **	0.303	−0.084	0.300
NH_4_^+^	−0.009	0.259	−0.104	0.124	0.204	0.090
Chl-a	−0.416 *	−0.552 **	0.027	0.295	0.282	0.007
TP	−0.322	−0.262	−0.093	0.540 **	0.311	0.254
TDP	−0.122	0.089	−0.156	0.126	0.261	−0.016

It has reported that MC production was positively related to trophic status and abundance of toxic *Microcystis* [45,46]. In northern lake areas, MC-LR and MC-RR were strongly negatively correlated to Chl-a (*p* < 0.05, *p* < 0.01 respectively) and have no clear relation with various forms of nutrients. The reason for high biomass of *Microcystis* whereas low relatively MCs might be due to a lower ratio of toxic to nontoxic strains [47]. In western lake areas, MC-LR concentration showed a positive correlation with TP (*p* < 0.01) and TN (*p* < 0.01), suggesting that nutrients favored *Microcystis* strains to produce MC-LR more than MC-RR in this area. It is important to note that the ratio of MC-LR to MC-RR in both parts of lake tends to be high in higher TN samples. Previous study demonstrated that MC-RR as nitrogen-rich variant was believed to be dominant in low C:N water [48]. Therefore, the result suggested the ratio of MCs variants determined by *mcy* genetic type [49] rather than nutrient concentration in Lake Taihu. The nitrogen concentration may influence the growth of certain genus *Microcystis* [50].

However, the study also showed that attention should be given to the risk associated with MCs during the period of cyanobacterial bloom in the summer. It is necessary to routinely monitor the MCs concentration during summer and autumn because they are very stable [6] and can enter the human body through the food chain. Additionally, water from Gonghu Bay and East Lake Taihu is used for drinking water. Because of the large number of aquatic plants and better water quality, Eastern Lake Taihu has a lower risk of MCs. Conversely, Gonghu Bay is adjacent to Meiliang Bay, where cyanobacteria blooms occurred frequently; accordingly, this region is at greater risk of MCs and should be subject to routine monitoring for MCs during summer and autumn. To better prevent the risk of MCs in the water source transferring from other heavy bloom lake areas, further studies should be conducted to construct a three dimensional coupled model that integrates hydrodynamics, water quality and ecological dynamics.

## 4. Conclusions

Based on monthly monitoring at nine stations from February 2009 to December 2011, Lake Taihu was in a hypereutrophic status in certain areas. Additionally, box plots indicated that various forms of nitrogen decreased annually from 2009 through 2011, which might have been related to non-point source pollution control in the watershed in recent years. The investigation showed that major nutrients, Chl-a and MCs (MC-LR, MC-RR) varied seasonally in the lake. Real-time data indicated unique characteristics of Lake Taihu at different stations that varied horizontally and vertically. As an important water source, MCs pollution of Lake Taihu is still serious. The control of nutrients would be an effective manner to reduce the risk of MCs, especially for the heavy bloom areas. This study provided a scientific foundation for the continuing water quality management of the Lake Taihu.
